# Occupation exposure to coal dust and vulnerability to Influenza: Scottish miners and the 1918 Influenza Pandemic.

**DOI:** 10.23889/ijpds.v9i5.2520

**Published:** 2024-09-18

**Authors:** Chris Dibben, Lee Williamson, Alice Reid, Eilidh Garrett, Peter Christen, Charini Nanayakkara

**Affiliations:** 1 University of Edinburgh; 2 University of Cambridge; 3 ANU

## Objective

There has been little work on the high level of mortality, noted that the time, in coal mining areas during the 1918 Influenza pandemic. Increased risk during viral infection from exposure to particulates (eg cigarette smoke, air pollution) has been studied.

## Approach

We use the historic administrative data for Scotland, 1900 to 1930, with standardised codes for occupation and cause of death, and a pseudo household design and Quasi Poisson models, where the spouses of miners are their controls, to test whether miners (aged 20 to 50) were more at risk of death during the main waves of the 1918 pandemic than a matched population. Secondly, we repeat the analysis for Labourers to test if [1] Sex or [2] hard labour and for clerks, if [3] Pulmonary Tuberculosis might explain the effect.

## Results

We find that miners compared to their spouses were significantly more likely to die during all waves of the pandemic but considerably more so during the Autumn wave (RR 4.7). This difference was even greater for younger miners (20 to 35) (RR 7.6). In contrast labourers and clerks had a much lower and often non-significant comparative risk of death during all waves than their spouses. This was true even for those aged 20-35.

## Conclusions

Male coal miners appear to have been at much higher risk of death compared to matched populations.

## Implications

Workers with exposure to airborne particulates should be a priority for public health planning during periods of high virus infections.

